# Design Is an Essential Medicine

**DOI:** 10.9745/GHSP-D-21-00332

**Published:** 2021-11-29

**Authors:** Pragya Mishra, Jaspal S. Sandhu

**Affiliations:** a Dalberg Design, Seattle, WA, USA.; b Gobee Group, Oakland, CA, USA.; c School of Public Health, University of California, Berkeley, Berkeley, CA, USA.

## Abstract

We provide an analysis of design in global health through a systematic framework to understand what it is, the value it can add, and how it compares to other common problem-solving approaches in global health. We make the case that design is an essential approach when tackling complex global health challenges.

## Key Messages

- Design as a practice is well placed to address the need to innovate faster, improve collaboration, and scale solutions while considering the way people live their daily lives across the world.
- Design for Health views design as a craft and a discipline that applies a specific mindset and skillset to a creative problem-solving process.
- The unique value of design in global health can be understood through 3 advantages: framing—identifying the right problems to solve; intention—creating space to solve problems the right way; and collaboration—continually engaging communities and organizations as actual partners.
- A comparison of design to other common problem-solving approaches in global health illustrates key differences but also offers opportunities to integrate design with approaches such as participatory research, quality improvement, and sociobehavioral research.
- To tackle complex challenges like the ones global health faces, there is a need for public health products, programs, and interventions to better meet the needs of communities. In the toolbox of approaches to global health innovation, design is essential.

## INTRODUCTION

The coronavirus disease (COVID-19) pandemic has revealed a few truths about global health: the speed of innovation needs to improve; we need to work more effectively across silos; and we need to understand that global challenges require solutions that take into account local cultures, social systems, and structures for them to be truly successful. There have been unprecedented leaps in biomedical innovation, particularly with drug and vaccine development.^1^ Despite challenges related to prevention and equitable access to resources, this pandemic has illustrated the capabilities of new innovation ecosystems, notably open innovation involving “purposive knowledge flows across organizational boundaries.”^2^

At once, the pandemic has unlocked new ways of collaborating and has exposed longstanding inequities in societies. Both of these are now a part of our working reality. This is an opportunity to radically rethink how we work in global public health, bringing with it the possibility to consider the role of design in this future world (Box 1).^3^

BOX 1 **What Do We Mean by Design?**

In this article, we use the term “design” as defined by the Design for Health community of practice:

Design is a craft and discipline that applies a specific mindset and skillset to a creative problem-solving process, enabling the development of informed, sensitive, inclusive, purposeful, appealing, and innovative solutions.^3^

The Design for Health community of practice, supported by the Bill & Melinda Gates Foundation and the Center for Innovation and Impact in the U.S. Agency for International Development’s Bureau for Global Health, includes global health practitioners, donors, nongovernmental organizations, design organizations, academics, and a cross-section of relevant experts from other disciplines that can help unpack the value design brings and how it can become a more integral part of global health. They state, “We embrace a broad definition of design, one that includes terms like human-centered design, service design, design thinking, and systems design.”

**FIGURE 1:**
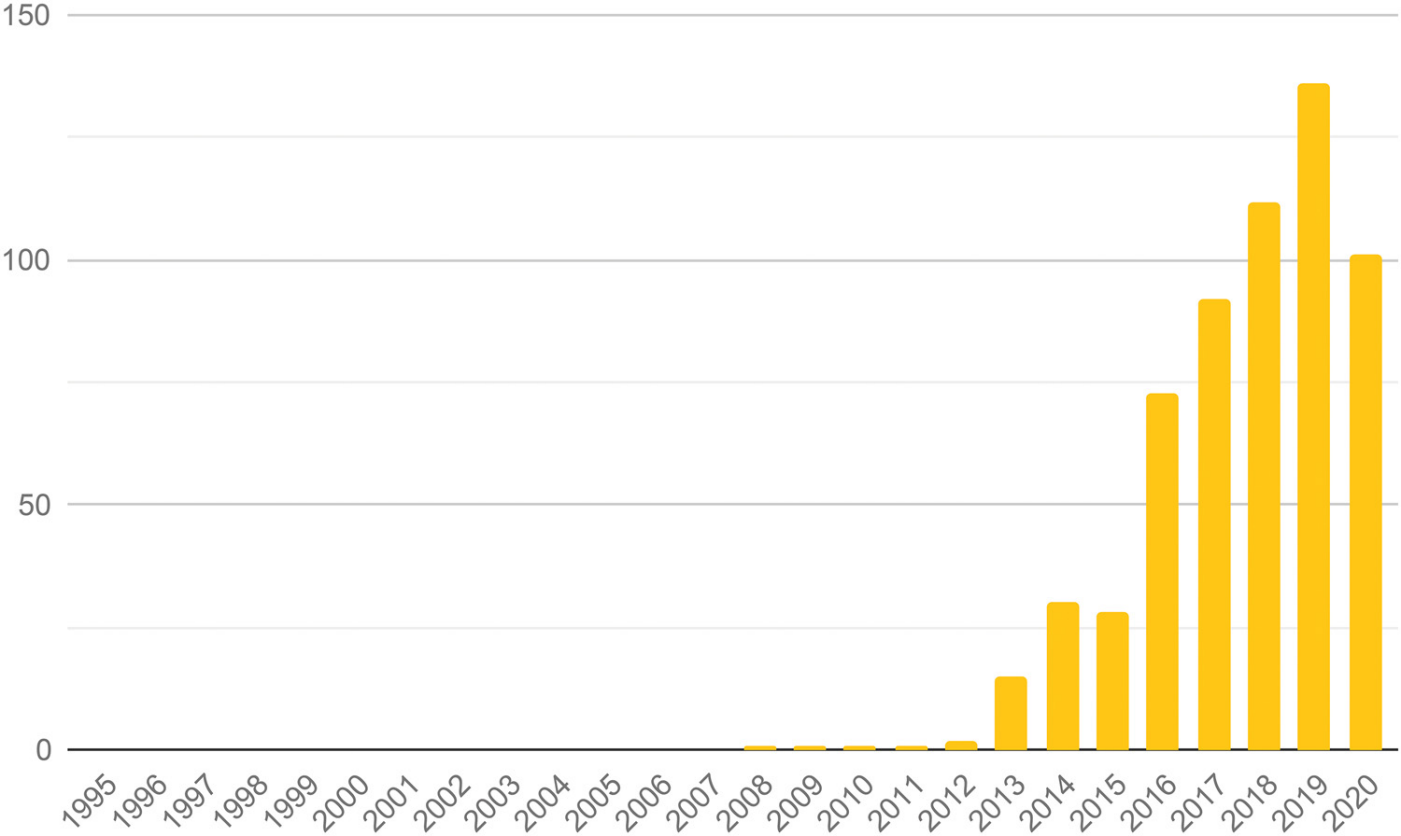
Documents by Year Containing the Phrase “Human-Centered Design” From the U.S. Agency for International Development’s Development Experience Clearinghouse^a^ ^a^ This represents approximately 625 documents or about 0.5% of the documents between 2008 and 2020. The decrease in 2020 may be due to the effects of COVID-19, bias in the dataset (documents from 2020 may not yet been included in the same intensity as prior years), or (less likely) a decrease in design-related activities at the U.S. Agency for International Development.

Design has increasingly gained recognition as a valuable approach to respond better to users’ needs and wants and to drive innovation. The Development Experience Clearinghouse (DEC) is a public repository of more than 200,000 documents from the U.S. Agency for International Development (USAID) spanning nearly 50 years.^4^ Given the scale of USAID’s involvement in global health and development, this database offers the opportunity to examine time-based trends for the work that governments and large global players have been doing over the same period. Searching DEC for “human-centered design” (HCD) illustrates both how design has been a part of global health for more than a decade and how it has become more pervasive over time (Figure 1).

Design has increasingly gained recognition as a valuable approach to respond better to users’ needs and wants and to drive innovation.

The natural compatibility between public health and design has been underappreciated during this time, deriving from common aims and values. Both design and global health are concerned with understanding complex and evolving systems. Both focus on groups or communities, rather than individuals. Consequently, both require an understanding of culture to be successful. Both are tasked with design decisions, often under considerable constraints, that must yield a substantial benefit. In this sense, both are concerned with the balance between cost and benefit and both require interdisciplinary collaboration.

As designers who have been working on different public health and global health challenges over the past 15 years, we believe that this is an important time for us to reflect on how design might and should function within global health. Design as a practice is well placed to address the need to innovate faster, improve collaboration, and scale solutions while considering the way people live their daily lives across the world. In this article, we examine what design means, how it can add value, and how it compares to other approaches in global health.

## DEFINING DESIGN

Design can be hard to understand because design and HCD are used to mean different things by different individuals, teams, and organizations. In many cases, definitions of design are based on individual interpretations of what design is and the role of designers as they view it. Bazzano et al.^5^ have discussed this in their scoping review of design in global health:

*No widely accepted definition exists within the broader design community on the essential characteristics that make [design thinking]/HCD different from other design or participatory practices … but rather varying definitions are used.*

Well before design or design thinking became common terms, experts from various fields—economics, academics, philosophy—articulated their ideas on creative problem solving. These ideas, in their essence, are a description of design thinking and process. In the mid-1950s, Buckminster Fuller created multidisciplinary design teams to tackle systemic failures. Fuller termed his approach, design science, as^6^:

*the effective application of the principles of science to the conscious design of our total environment in order to help make the Earth’s finite resources meet the needs of all of humanity without disrupting the ecological processes of the planet.*

In 1958, the government of India invited Charles and Ray Eames to advise on the creation of a design institute to serve local small industries. Their recommendations took the form of “The India Report,”^7^ in which they expressed the desire for an institute that trained individuals to tackle challenges by adopting an incremental problem-solving attitude. They illustrated this idea through the design of a lota, a simple vessel used in many Indian households. They noted that the lota had been perfected over generations with many individuals adding refinements, carefully considering 1 factor after the other over time—the optimum amount of liquid to be fetched, carried, poured, and stored in a prescribed set of circumstances, the possible materials of production, and the costs involved.

In 1969, Nobel laureate Herbert A. Simon described design as a science or way of thinking in his book, *Sciences of the Artificial*^8^:

*Everyone designs who devises courses of action aimed at changing existing situations into preferred ones. The intellectual activity that produces material artifacts is no different fundamentally from the one that prescribes remedies for a sick patient or the one that devises a new sales plan for a company or a social welfare policy for a state.*

## UNDERSTANDING THE DESIGN PROCESS

As for the process, researchers Don Koberg and Jim Bagnall, in their book from 1972, The *Universal Traveler: A Soft-Systems Guide to Creativity, Problem-Solving, and the Process of Reaching Goals,* used the analogy of traveling to describe a systematic approach to problem solving. They described a problem solver starting the journey by accepting a situation then moving to analyzing, defining, ideating, selecting, and implementing to end the journey at evaluating.^9^

These early interpretations of design processes have been more recently adopted and adapted by organizations and practitioners working in varied sectors, bringing with them their own practical experiences and unique ways of working within their organizations. Even the same organizations have often iterated on this process as their own practice has evolved from applying design methods to products, services, and now to complex systemic issues. At any given moment, an organization might have different ways of expressing its design process. We examine a few of these well-established models to better understand the various ways in which people have expressed the way that they think about design processes.

**FIGURE 2:**
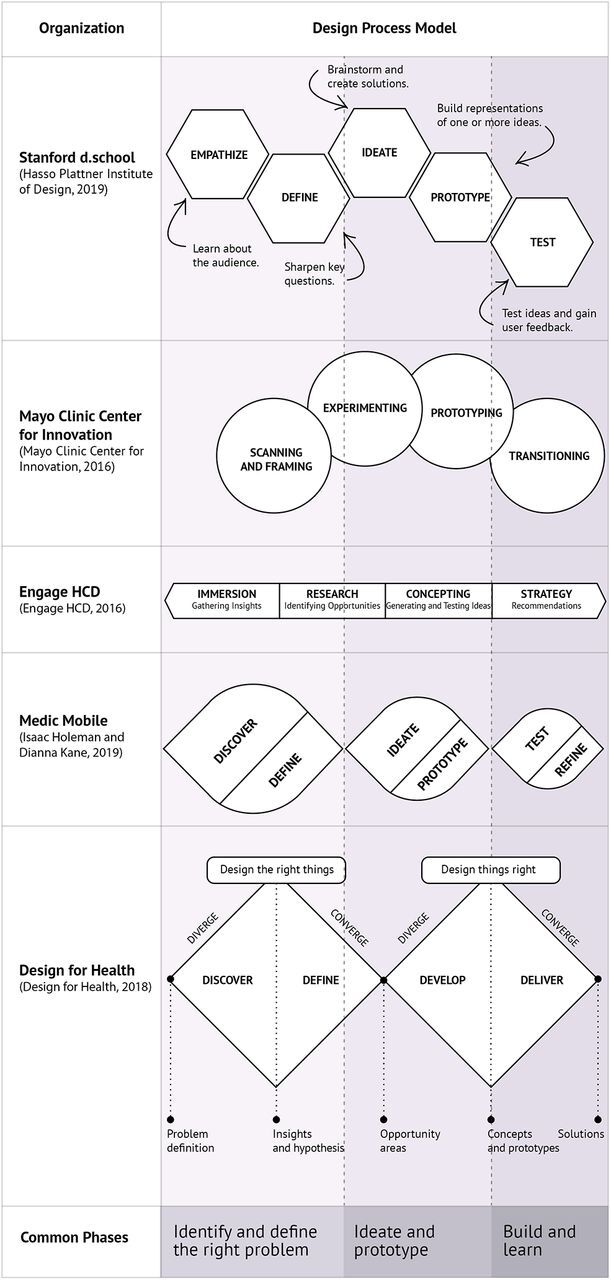
Comparison and Synthesis of Selected Design Processes^a^ ^a^ Many other prominent models of design exist. We have chosen these 6 illustrative models to represent a breadth of organization types, and we have intentionally oversampled models from health care, global health, and global development. These models are from Hasso Plattner Institute of Design,^10^ Mayo Clinic Center for Innovation,^11^ EngageHCD,^12^ Medic Mobile,^13^ and Design for Health.^3^ The authors are contributors to both EngageHCD (PM) and Design for Health (PM, JS).

At a glance, these design processes may seem unique, but a closer inspection reveals more similarities than differences (Figure 2). They begin with research followed by iterative cycles of ideation and prototyping to eventually converge on the most promising solutions.

Design processes begin with research followed by iterative cycles of ideation and prototyping to converge on the most promising solutions.

What value do the numerous variations of process add? Sometimes, there is not much difference. This has happened in other fields, such as quality improvement (QI). Walshe^14^ describes many competing QI approaches as appearing to be different but having underlying similar approaches.

A closer look at Figure 2 shows that these selected processes begin to diverge in the concluding phases that focus on outcomes. The variations in each of these models reflect their adaptation to the needs of a specific organization or context. Their value lies in the fact that they are customized for specific purposes. For example, the Mayo Clinic’s Center for Innovation defines its final phase as “transitioning.” This is appropriate in their context where, as an in-house consultancy, they need to find the right team to own the outcomes and take them forward. But in the case of Medic Mobile which owns its outputs, they continue to “test” and “refine” ideas and develop them further as they implement and learn from them (Figure 2).

## THREE ADVANTAGES OF DESIGN

**FIGURE 3:**
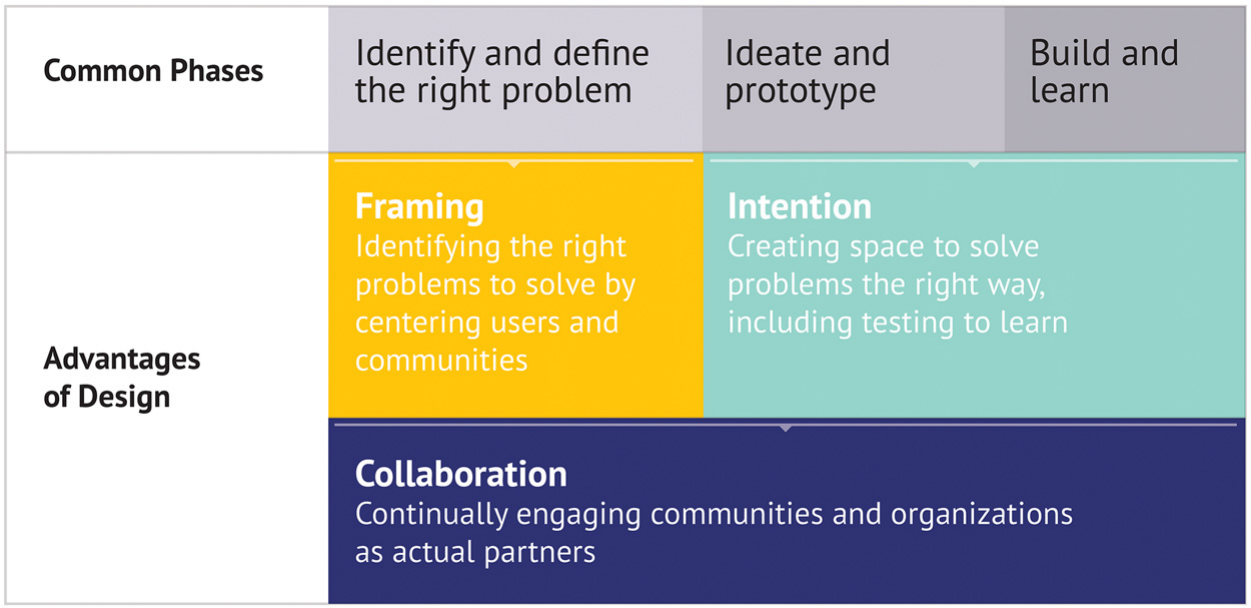
Common Phases Seen in the Design Processes Mapped to the Advantages of Design

While an exploration of process can clarify what design is, it does not speak to how and why design can be beneficial to global health. However, each step of the design process brings clear advantages (Figure 3).

1. **Framing:** Design identifies the right problems to solve by keeping the user and community perspectives at the center. In the early stages of the design process, designers conduct research with different stakeholders in the ecosystem to gain a better understanding of a problem and the context in which it exists. This helps in designing the right things that are tailored to the needs of the community that the designers are working for.
2. **Intention:** Design creates space to solve problems the right way, including testing to learn. The deliberate and thoughtful process of co-creating different ideas and testing them with communities, early and often, to eventually converge on the most effective solutions leads to designing things the right way, respecting the experiences, wants, needs, and priorities of communities. Such an approach that intentionally accounts for the needs of users and communities at each step of the process can help avoid potentially costly mistakes during implementation.
3. **Collaboration:** Design continually engages communities and organizations as actual partners throughout the design process. The iterative nature of design relies on continued inputs from the communities that designers are working for and from stakeholders such as technical experts from other disciplines they are working with. The true value of design is fully realized when multidisciplinary teams work with communities to identify their needs, co-create and test ideas, and facilitate decision making. This type of collaborative design process can lead to more sustainable and equitable outcomes because it takes into account how people live and respects their wants, needs, and cultural beliefs.

Design identifies the right problems to solve by keeping the user and community perspectives at the center.

Within global health, as the demand for design has grown, practitioners have grappled with how to define design, effectively apply it to their work, and demonstrate its impact. To help address this, in 2017, the Bill & Melinda Gates Foundation and the Center for Innovation and Impact in USAID’s Bureau for Global Health established Design for Health, a design-focused community of practice. Design for Health is how we as authors first came together. We also believe that the Design for Health framing has exceptional validity because so many organizations inside and outside of design have negotiated it. A notable analog to Design for Health is the Innovation Learning Network, which recognized the value of “coopetition” in sharing approaches to innovation IN U.S. health care.^15^ Design for Health views design as a craft and a discipline that applies a specific mindset and skillset to a creative problem-solving process. The design mindset focuses on engaging people early and throughout the process of developing solutions. Design also applies different skills to specific challenges, across different project stages. Individual designers typically possess a depth of knowledge in one or more design areas. Like all health care professionals who have a basic understanding of clinical practice, designers possess common skills in creative problem solving, visual thinking, and the craft of making things.^3^

As demand for design has grown, global health practitioners grapple with how to define design, apply it to their work, and demonstrate its potential impact.

**FIGURE 4:**
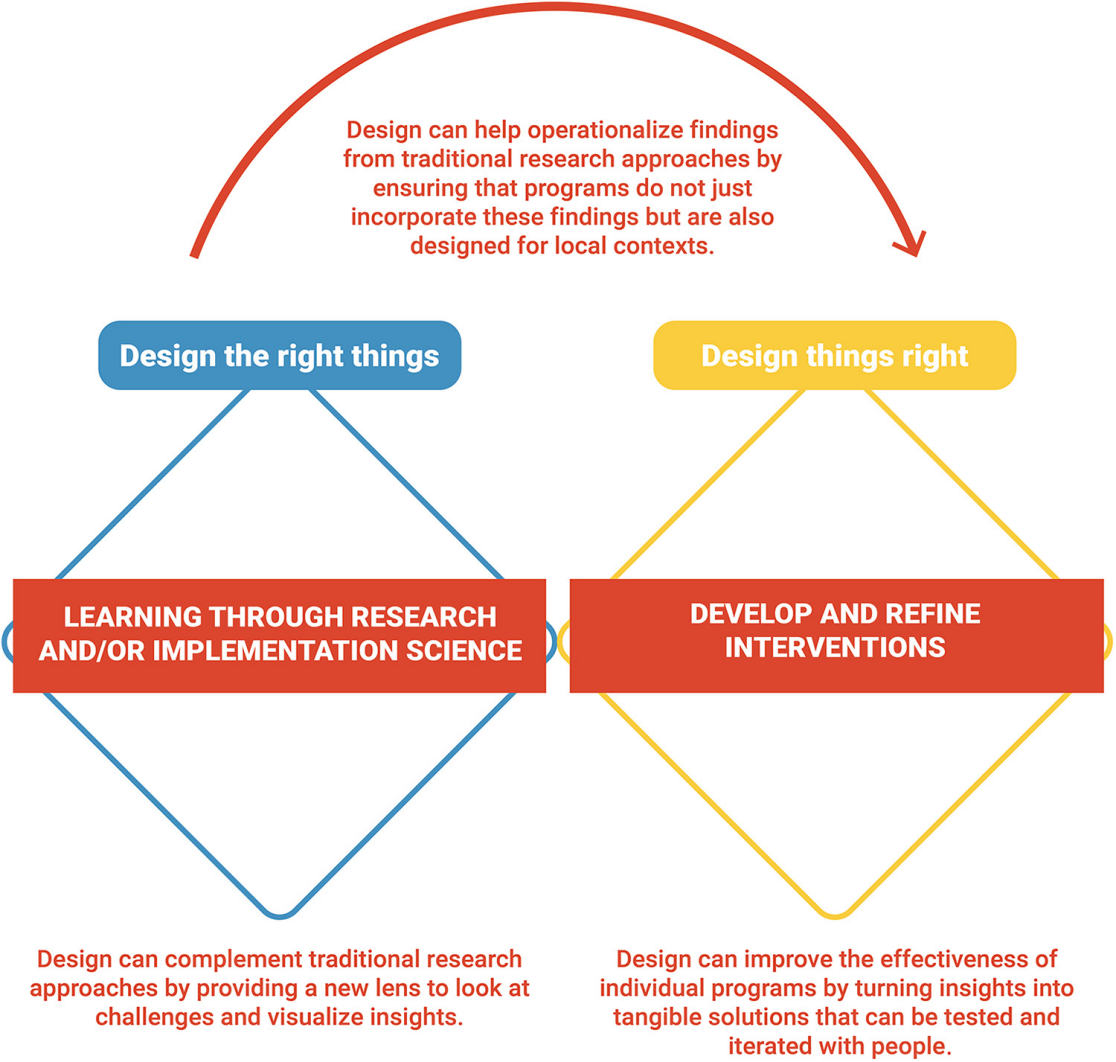
The Design Process in Global Health^a^ ^a^ As described by Design for Health.^3^ The first cycle, which helps design the right things, starts with discovering the challenges and needs of the people and systems to design for and converge on opportunities to overcome these challenges and address these needs. The second cycle helps design things right by developing and testing ideas based on the opportunities identified in the first cycle and then converging on and delivering a solution. The model emphasizes testing and iteration, which helps weed out ineffective ideas and prioritize impactful, desirable, and sustainable ideas.

While each design specialization—such as visual design or product design— has a different way of working, the creative process of design shares commonalities across specializations. Design for Health adopted the Double Diamond model^16^ to illustrate these commonalities in the design process while mapping it to the way the global health community develops solutions—through research or implementation science and using the findings to develop and refine interventions that address global health challenges (Figure 4).^3^

## THE VALUE OF DESIGN IN GLOBAL HEALTH

Global health typically deals with complex problems, involving numerous stakeholders working within long-established systems. In most cases, it also means working with limited budgets and resources that need to be used judiciously. These systems also bring with them systemic inequities based on ethnicity, race, gender, education, income, class, disability, geographic location, and sexual orientation that have been built in over time. These inequities, unless intentionally addressed, are reinforced or exacerbated. This makes it critical that the measure of success of any intervention today considers efforts made to advance equity. Successful interventions in global health also require breaking down complexity, innovative thinking, and the ability to give voice to diverse perspectives while working with limited resources. So, in this context, how can design add value?

**FIGURE 5:**
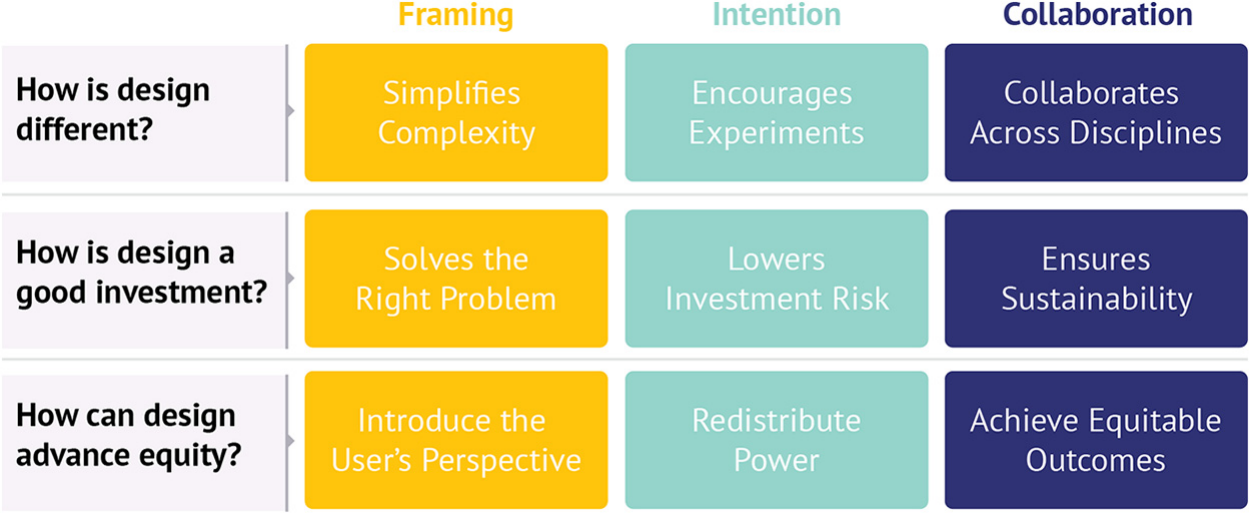
Nine Benefits of Design Organized by the 3 Defining Advantages of Framing, Intention, and Collaboration^a^ ^a^These questions are derived from common questions about design that we and our colleagues have fielded from global health practitioners. Together, these benefits describe the value of design.

The defining advantages that we outlined in the previous section: framing, identifying the right problems to solve; intention, creating space to solve problems the right way; and collaboration, continually engaging communities and organizations as actual partners also speak to the value of design. To examine the value of design further, we use these advantages to answer 3 questions that global health practitioners often ask us (Figure 5): (1) How is design different? (2) How is design a good investment? (3) How can design advance equity?

### How Is Design Different?

The benefits of using any approach can at least partly be attributed to its unique aspects. In the case of design in global health, design helps to frame complex issues simply, be intentional when experimenting with a range of possible solutions, and support meaningful collaboration across communities and disciplines.

Design in global health helps to simplify complexity, encourage experiments, and collaborate across disciplines.

#### Simplifies Complexity

Mani-Kandt and Robinson^22^ found that successful HCD efforts in international development usually have a narrow problem or focus. Over the past 5 years, our respective work has explored complexity more deeply. Problems do not need to be narrow at the start. Design helps in breaking down daunting, complex issues into more tangible ones that are easier to analyze and solve. By using techniques such as data visualization, design can help surface patterns in the data that can otherwise be hidden, tell a compelling story by emphasizing key insights, and help make better decisions by focusing on what is most important. For example, designers often use ecosystem maps and customer journey maps to effectively communicate complex systems by identifying the various stakeholders involved, the relationships that they share, and the bottlenecks in the system that need the most attention.

#### Encourages Experiments

Design tools and methods like ideation and iterative prototyping allow teams to quickly and easily test and build on ideas, pivot toward new ones, and make decisions with the users in a manner that gives a sense of assurance at each stage of a project. This means that teams can move more confidently from their initial introduction to an issue to exploring and experimenting with a range of possible solutions; surfacing new channels, touchpoints, and influences; and eventually converging on those solutions that show the most promise. This work often involves co-creation of prototypes with a core team and direct testing with users, as Paper-based Health Information Systems in Comprehensive Care did in developing tools to support decision making for health care workers in Côte d’Ivoire, Mozambique, and Nigeria.^23^ These co-creation processes yield out-of-the-box thinking by engaging both designers and non-designers in the creative process. Meanwhile, direct testing with users provides rapid feedback and validation before moving to more extensive studies or direct implementation.

#### Collaborates Across Disciplines

Design supports a multidisciplinary approach to problem solving that can surface new perspectives and connect disparate insights across complex health systems. In a graduate-level course that one of the authors taught for 8 years—the first HCD course in public health—the first 100 students came from 26 distinct academic programs.^24^ This type of diversity can lead to innovative solutions that otherwise might have been overlooked. Designers tend to collaborate with each other, different disciplines, and communities to generate ideas, test hypotheses, and create products and strategies. This cross-pollination can be a path to innovation as global health expertise combines with creative approaches and other disciplines.

### How Is Design a Good Investment?

Often the scale of the challenges in global health combined with the fear of making costly mistakes results in teams being overly focused on either preventing errors or relying on tried and tested approaches, rather than on exploring new and innovative ways of tackling an issue. Design addresses this by focusing on the right problem to solve and reframing these challenges in new ways when useful. It lowers investment risk by prototyping, testing, and iterating solutions with communities and by ensuring stakeholders have real ownership of outputs making the solutions more resilient and sustainable.

Design addresses efforts to prevent errors by focusing on the right problem to solve and reframing these challenges in new ways when useful.

#### Solves the Right Problem

Individuals, teams, and organizations can be highly effective at solving problems together. Unfortunately, within and outside of global health, global health practitioners are often tasked with solving problems that do not necessarily address the needs, priorities, and wants of the communities they hope to solve for. Design not only provides an opportunity to understand old challenges in new ways but works with communities to identify the right problems that need to be solved. A significant aspect of a design effort may be focused on reframing a challenge where the question that a team starts with may not be the one that it arrives at. In Pathways, a portfolio of projects supported by the Bill & Melinda Gates Foundation, work with multiple design partners provided a novel understanding of the different risks, barriers, and access that women and girls face “in seeking improved health.”^25^ Prior thinking was centered on biological and clinical perspectives and did not account for individual differences in risks, barriers, and access. This social vulnerability framework “reframes questions from a human-centered view,” elevating the real-life social and environmental influences that shape the unique experiences of women and girls. The Pathways framework has since provided input into Bill & Melinda Gates Foundation’s thinking related to maternal and child health. In reframing, as Pathways has done, the new question will consider the key priorities of the community, incorporate lessons from what has been tried, and scope the problem in a manner that also aligns with the existing strategies in global health.

#### Lowers Investment Risk

By listening to the community; using prototypes to test, fail fast, learn, and iterate with them; and empowering them to make decisions, design can reduce the time, effort, money, and other resources necessary to tackle a problem. Continually engaging the community and co-creating and validating ideas early and often through an iterative process improves the odds of developing solutions that are not only more innovative but can also reduce the risk of making big costly mistakes. An IBM study determined that its internal design thinking practice achieved a 301% return on investment through improved product outcomes, increased average product profits, and reduced risk of costly failures.^26^ As Cherney et al.^27^ wrote:

*As an added benefit, this approach increases confidence that chosen approaches will be accepted by their intended users at and long after launch.*

Continually engaging the community and co-creating and validating ideas early and often improves the odds of developing solutions that are more innovative and reduce the risk of making big costly mistakes.

#### Ensures Sustainability

Engaging the right group of stakeholders in the design process can lead not only to better innovation but also to real ownership over the product, program, service, or policy. This ownership is an important factor in the long-term sustainability of any solution to create “solutions that stick.”^24^ Every solution will need to be implemented, supported, monitored, and improved over time. Adopting an approach that gives stakeholders a sense of ownership over solutions leads to higher uptake and hence more resilient and sustainable solutions.^28^

### How Can Design Advance Equity?

Inequities that exist in societies also manifest themselves in health care systems. At an institutional level, inequity can be supported by policies and practices, and at an individual level, they can take the form of unconscious bias. Such disparities can be hard to recognize and identify but have real and grave consequences for those who face them. Design can help advance equity by framing problems from a user or community perspective, intentionally redistributing power and shifting it toward communities, and collaborating with communities to enable outcomes that are also equitable.

Design can help advance equity by framing problems from a user or community perspective, intentionally redistributing power and shifting it toward communities, and collaborating with communities to enable outcomes that are also equitable.

#### Introduce the User’s Perspective

Design helps view problems, products, or services more holistically through a health systems lens rather than viewing them through expertise or program silos. Bringing the focus to a user’s experience with a product or service helps teams prioritize and design solutions that consider the users’ experiences, wants, needs, motivations, and behaviors, as well as other local cultural factors. In their Demand for Health Services Toolkit, United Nations Children’s Fund (UNICEF) describes the central importance of people^29^:

*Health programs are people programs. At every step of the way they involve people, from government officials to community health workers. Perhaps nowhere is the involvement of people more important than with users, or the people for whom programmes exist.*

It may sound obvious to include the perspectives and voices of users, but the continuous inclusion of users is not the norm in global health. Design offers a philosophy and framework for doing this. Solutions that meaningfully engage the user’s perspective can better fit into the context of users’ day-to-day lives and lead to better uptake and sustained use.

#### Redistribute Power

One of the primary ways that design can enable equitable outcomes is by revealing existing inequities, questioning biases, and enabling the integration of community members affected by inequality and oppression as lived-experience experts to be part of the design and decision-making process. Design can facilitate the continued engagement of the community in the project process that goes beyond them being input providers rather than active participants in solving a problem. One example is the place-based initiative Best Babies Zone that sought to reduce inequities in infant mortality by engaging community innovators in leading the design process by both defining priorities and solving problems.^30^ With such efforts, the power shifts from the “benefactor” (and a benefactor-led agenda) toward community-driven priorities that are guided by local needs, aspirations, opportunities, and outcomes.

#### Achieve Equitable Outcomes

Design, when used well, can be a tool that empowers people to make decisions and not simply relegate them to being passive consumers of solutions that “experts” have developed. While design can enable equitable outcomes, designers should also be careful to ensure that it does not end up being another method by which existing power hierarchies are perpetuated further. By reducing the reliance on “efficiency” as the primary metric of measuring success and instead focusing on empowering communities to articulate their problems, generate solutions, and make decisions on the right solutions, design can facilitate better outcomes. It is equally important, and this goes well beyond design, that we work to support the most marginalized and least visible groups.^29^

In our experience working in global health, we have seen design bring these benefits to projects and initiatives when it has been set up to succeed. This means that as global health practitioners, along with acknowledging the value of design, we need to commit to positioning design on an equal footing with other disciplines. Design can provide value in global health, but we do not consider it to be the only approach available.

## COMPARING PROBLEM-SOLVING APPROACHES

We expect that readers of this article will have varying levels of familiarity with design. At the same time, we expect most readers to have some exposure to approaches that may share purpose or techniques with design. This provides us with the opportunity to describe design by understanding its relationship to these other problem-solving approaches.

In this universe of approaches to problem definition and problem solving, some may be considered complementary approaches to design (e.g., behavioral economics and data science) while others may be seen as alternative approaches that achieve similar ends. This is a fuzzy boundary. Additionally, while some are considered disciplines, others might be considered approaches or methods. Comparing these approaches is a complex, messy, and fraught exercise, which may explain why it has not been done comprehensively before. Pairwise comparisons have been made, but there remains a need to see the bigger picture. We are engaging in this activity precisely because there are limited comparisons like this in the literature. We recognize the danger in simplifying approaches for this comparison but believe the value to the global health community is paramount.

**FIGURE 6:**
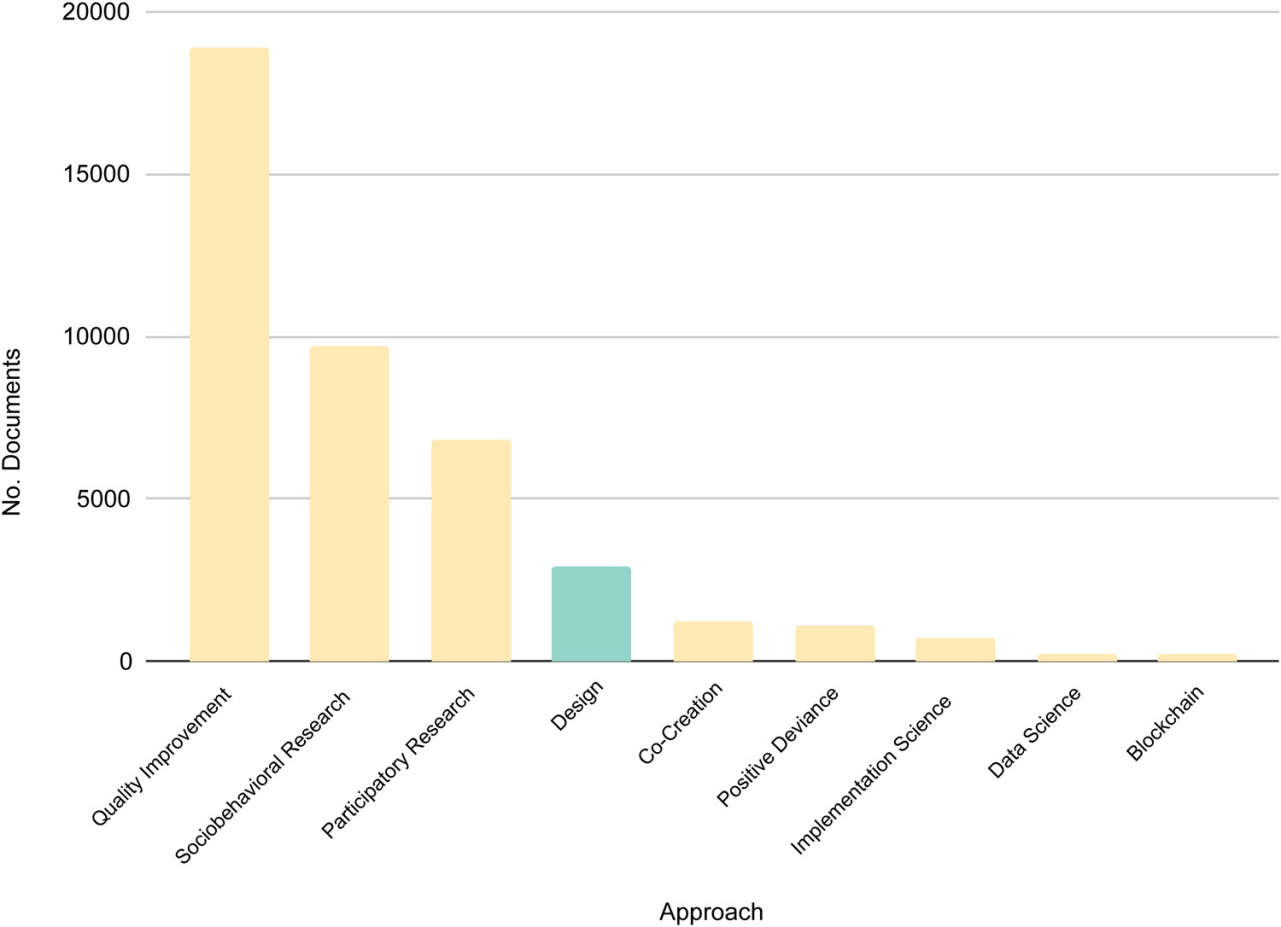
Document Frequency of Key Approaches in the U.S. Agency for International Development’s Development Experience Clearinghouse, 1975–2020^a^ ^a^ For each approach, we conducted a keyword search over all the text of all documents, including synonyms, unique abbreviations, or adjacent approaches when appropriate.

In our analysis, we compared design to 3 broad approaches used in global health: participatory research, QI, and sociobehavioral research (Table). We decided to focus on these based on further exploration of the USAID DEC database (Figure 6).

**TABLE.** Design Compared to 3 Approaches in Global Health

	**Participatory Research**	**QI**	**Sociobehavioral Research**	**Design**
Related terms and approaches	CBPR Participatory action research Youth participatory action research	Lean QI Continuous QI Performance improvement TQM PDSA Six Sigma	Qualitative research Social-behavioral research Formative research Cultural anthropology	HCD Design thinking User-centered design
Framing	Partner with communities to define the problems that matter to them	Understand problems in context of existing systems and subsystems	Use qualitative research methods, sometimes ethnographic approaches; formally approach sampling, recruitment, data collection, analysis	Understand problems in context; use methods from qualitative research, with flexibility to adapt approaches
Intention	Generate research for future action; develop localized ownership and solutions	Improve existing systems using a continuous approach to testing and measurement	Provide inputs to program design or general knowledge	Fundamentally innovate, through creative processes and prototyping; sometimes improve existing systems
Collaboration	Partner with community members, establish long-term relationships	Identify teams within an existing organization or system	Create qualitative research teams, who sometimes immerse in a group or culture	Users may be partners, participants, or subjects in design; engagements are days or weeks, not months or years
Outputs	Research; community ownership of research; community capacity building	Measurably improved processes within existing systems	Peer-reviewed research; ethnographic accounts; program recommendations	Innovation in form of service, product, strategy
Citations and further reading	Chen et al.^32^ Kia-Keating et al.^33^	Kachirskaia et al.^34^ Ahn et al.^35^	Tolley^36^ Design for Health, 2019 Complementary Approaches^37^	-

Abbreviations: CBPR, community-based participatory research; HCD, human-centered design; PDSA, plan-do-study-act; QI, quality improvement; TQM, total quality management.

**Figure:**
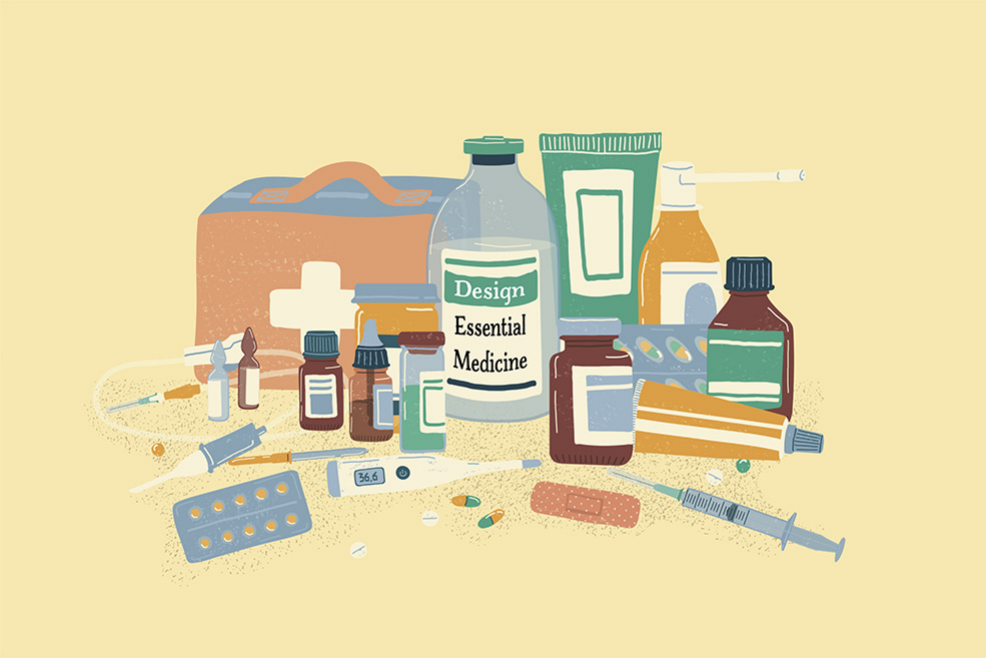
Design is an essential medicine. © Vector Point Studio/Shutterstock (modified)

These 3 approaches appeared in higher numbers than design-adjacent terms. Other candidates that we had considered, such as implementation science and positive deviance, do not appear as frequently, so we have omitted them from our comparison. This should not be interpreted as a judgment on their value. We have included related approaches when possible. This is not to suggest that they are all the same. In fact, there are discussions in many of these fields, as there are in design, to differentiate between approaches (e.g., lean versus QI).^31^ However, there are similarities between design and these other approaches, which can confuse global health practitioners who are not immersed in any of these approaches. We aim to provide a sufficient description to allow the reader to understand critical differences among the families of approaches. It is not exhaustive.

## THE PATH FORWARD: INTEGRATING APPROACHES

As design matures in public health, health care, and global health, we recognize an emerging pattern: design and nondesign practitioners alike see the opportunity to integrate design with other approaches.

We recognize an emerging pattern: design and nondesign practitioners alike see the opportunity to integrate design with other approaches.

Chen et al.^32^ identify 5 specific approaches that community-based participatory research can borrow from design in developing a new project, including centering empathy and rapid prototyping. Kia-Keating et al.^33^ have integrated community-based participatory research and HCD to address health disparities related to violence among Latinx youth in the United States. Notably, they adopted design-based approaches to idea generation. Kachirskaia et al.^34^ have discussed Kaiser’s approach to “fusing” performance improvement and HCD:

*Using HCD generates deeper engagement in PI efforts among Kaiser Permanente patients and family members, who have expressed positive experiences of being involved in projects using HCD.*

Ahn et al.^35^ conducted a study of health care leaders in the United States focused on the role of Lean in breakthrough improvement but found an unanticipated result:

*the value of [HCD] thinking, alone or as a complement to Lean management, in achieving breakthrough improvement in health care organizations.*

Tolley^36^ poses the question directly in considering sociobehavioral research and design:

*Is it possible to draw on the strengths of each strategy to enhance user-centered research more generally?*

This suggests that continued blending of approaches will occur in global health and beyond. So, in addition to our observation that it **is** happening, we argue that it **should be** happening. It will be critical to understand and retain the unique values of different approaches in doing so; ensure that the methods and approach match the problem; and ensure that the approaches used have the appropriate time, space, and support to achieve successful outcomes to not be prematurely dismissed.

Integrating approaches may accelerate the inclusion of design in the toolkits of different organizations. Experiences with the Aravind Eye Care System^17^^–^^21^ (Box 2) and findings from ITAD^22^ suggest that some organizations will be more equipped than others to adopt new approaches like design. Adaptive mindsets, supportive leadership, and flexible management are all keys to a more ready adoption of design.

BOX 2 **Aravind: Embracing Design Mindsets and Approaches for 45 Years**

We highlight the example of the Aravind Eye Care System to illustrate how the mindsets and principles of design are found within global health already if one only looks in the right places. Aravind, based in Madurai, Tamil Nadu, India has long been the global pioneer in providing no-cost and low-cost care to treat blindness. Founded in 1976 by Dr. V (G. Venkataswamy) and his family members, a group consisting of ophthalmologists, engineers, and managers, Aravind has now been innovating for nearly half a century. Today, they perform 500,000 surgeries annually, along with more than 4.5 million outpatient visits. Their clinical outcomes across these incredible patient volumes are so outstanding, that researchers, health care administrators, and clinicians have been making the learning pilgrimage to Madurai for decades. While Aravind did not explicitly frame their work as design, they have embraced the spirit of design in innovating services, products, and an entire health care system.^17^^–^^19^ Since its inception, Aravind has expanded its direct services from cataract surgeries to all aspects of ophthalmic treatment and prevention. They have constantly innovated in service delivery, clinical workflows, financial models, technology for care provision, and product development. They have exported low-cost ophthalmic products that they have developed to more than 130 countries through their product development company Aurolab. Through the Lions-Aravind Institute for Community Ophthalmology (LAICO), they have provided technical assistance to more than 350 eye hospitals in more than 30 countries and trained eye care professionals from 80 countries^20^; (T. Ravilla, personal communication, August 30, 2021).

One central design mindset that Aravind has used across the organization and throughout its history is learning by doing.^21^ Consider the case of the original eye screening camps that Aravind developed in the late 1970s. The compliance was less than 20%, meaning that less than 1 in 5 potential patients took advantage of the offer of free surgery. During these camps, staff learned of the many barriers that people in poverty and living in rural areas experience, including food, lodging, and transportation. These barriers should not be a surprise to those who work in global health, but it is how Aravind responded that sets them apart. Through a process of experimentation, they added services to directly address those barriers and increased yields to more than 90%. The learning-by-doing mindset is incomplete if it does not include a willingness to learn from unsuccessful experiments. Aravind did this, too. In the 1980s, they conducted surgeries in rural makeshift facilities. They could not achieve the quality of outcomes that define the organization, so they abandoned this strategy and redoubled efforts to connect rural patients to their centralized hospitals.

Aravind has worked continuously to understand the true barriers to accessing care (framing), they have tested and implemented new approaches that diverged from existing models (intention), and they have worked in deep partnership with the community to uphold their values of service to others (collaboration). Aravind’s efforts highlight the possibility of design, especially when paired with a focus on systems and scale and embedded in a mission-driven organization. Aravind has exemplified that design can be embedded within global health organizations and suggests that there is real potential to further democratize design.

There are other approaches that we have not discussed that often come up in our conversations about design in global health. Some of these are alternative approaches, some are complementary, and some fall in between. These approaches include user experience, positive deviance, systems thinking, collective impact, implementation science, social and behavior change, co-creation, market research, behavioral economics, and data science. Pairing design with other approaches such as these will unlock its greatest potential, as Johnson et al. wrote^25^:

*Integrating design with complementary disciplines … amplifies the impact of design, providing a new lens into how the field of global health has traditionally designed solutions to some of its most intractable problems. However, this requires openness to new lessons from all the participants, and they must be prepared to build on them.*

Exploring how design might be integrated with various other approaches is beyond the scope of this article. We can say that this integration is critical, not for design as a discipline, but rather for global health as a whole. Integration will ensure problem-solving approaches that match the challenges, stronger collaboration among global health practitioners, and a faster, more cost-effective path to people-centered innovation.

Integrating design and global health approaches will ensure problem-solving approaches that match the challenges, stronger collaboration among global health practitioners, and a faster, more cost-effective path to people-centered innovation.

## CONCLUSION

In 1977, when the World Health Organization published its first essential drugs list,^*^^,^^38^ now known as the Essential Medicines List (EML) with 212 medicines, it was hailed as a "peaceful revolution in international public health."^39^ Today, the EML includes 460 medicines and 80% of countries have a national essential medicines list based on the WHO EML. While many differences exist among these lists,^40^ there is a common global approach to prioritizing and securing evidence-based medicines. This has enabled improved supply, higher product quality, improved cost management, and a higher quality of care.^41^

What has become increasingly apparent during these last 50 years is the parallel need for public health products, programs, and interventions to better meet the needs of communities. At times, an overemphasis on supply and access has ignored demand, which is rooted in the real, complex lives of people and families. A host of different approaches to innovation have emerged during the era of the EML, from social marketing to public-private partnerships to root-cause analysis. These are indispensable to global public health, but gaps remain in how we address global health challenges. These gaps are related to how people actually behave instead of how we think they should behave. COVID-19 has emphasized this disconnect. In the toolbox of approaches to global health innovation, design is critical. This toolbox is an essential processes list, and design must be on the list.

While the COVID-19 pandemic and our response to it have surfaced opportunities for design to be used more within the global health sector, the reality is that, for years and decades, there has been an abundance of pressing issues—climate change, urbanization, information epidemics, infectious disease, poverty, and inequity—that have continued to exert pressure on traditional approaches to global health challenges. It is incumbent on us as global health practitioners that we heed these warnings and reflect on how we can make a difference. Design is an essential medicine for global health at this critical inflection point, so its practitioners must understand its indications for use.

## Footnotes

* Cuba was the first nation to create a basic medicines list in 1963, followed by Tanzania (1970) and Peru (1972).

## Notes

**Peer Reviewed**
